# Tuning reductase activity in monoterpene indole alkaloid biosynthesis

**DOI:** 10.1073/pnas.2605425123

**Published:** 2026-06-15

**Authors:** Samuel C. Carr, Song Wu, Klaus Gase, Yoko Nakamura, Mohamed O. Kamileen, Maritta Kunert, Sarah Heinicke, Moonyoung Kang, Veit Grabe, Lorenzo Caputi, Sarah E. O’Connor

**Affiliations:** ^a^ Department of Natural Product Biosynthesis, Max Planck Institute for Chemical Ecology, Jena 07745, Germany; ^b^ Microscopic Imaging Service Group, Max Planck Institute for Chemical Ecology, Jena 07745, Germany

**Keywords:** natural products, biosynthesis, alkaloids, heterodimer, geissoschizine

## Abstract

Monoterpene indole alkaloids encompass a diverse class of plant natural products. Many of these valuable compounds, including the anticancer medicines vinblastine and vincristine (*Catharanthus roseus*) and the antiaddiction treatment ibogaine (*Tabernanthe iboga*) utilize a common biosynthetic intermediate, 19*E*-geissoschizine, which is produced by the medium-chain dehydrogenase/reductase geissoschizine synthase (GS). Herein, we report facilitator of geissoschizine synthase (FoGS), a protein that improves formation of 19*E*-geissoschizine by acting in combination with GS. The identification of a FoGS ortholog that works with GS to produce 19*Z*-geissoschizine shows how this protein also enables control over product stereochemistry. We demonstrate that FoGS and GS interact to form a stable heterodimer and through mutagenesis identify FoGS residues that contribute to the combined activity of FoGS and GS. This work highlights the involvement of proteins in metabolic pathways that, when assayed alone, appear nonessential, but nevertheless increase the efficiency or specificity of a given metabolic step.

## Significance

Monoterpene indole alkaloids are a widespread group of plant derived metabolites with a broad range of biological and pharmacological activities. A key step in monoterpene indole alkaloid biosynthesis is catalyzed by the enzyme geissoschizine synthase (GS). Here, we identify partner proteins that influence the stereoselectivity and increase the overall efficiency of GS. We show that these partner proteins and GS interact, likely forming a heterodimer. Since GS is required for production of medicinally important monoterpene indole alkaloids (e.g., vinblastine, an anticancer agent, and ibogaine, a psychoactive), these partner proteins may be important for improved access to these high value compounds through metabolic engineering.

**Fig. 1.**
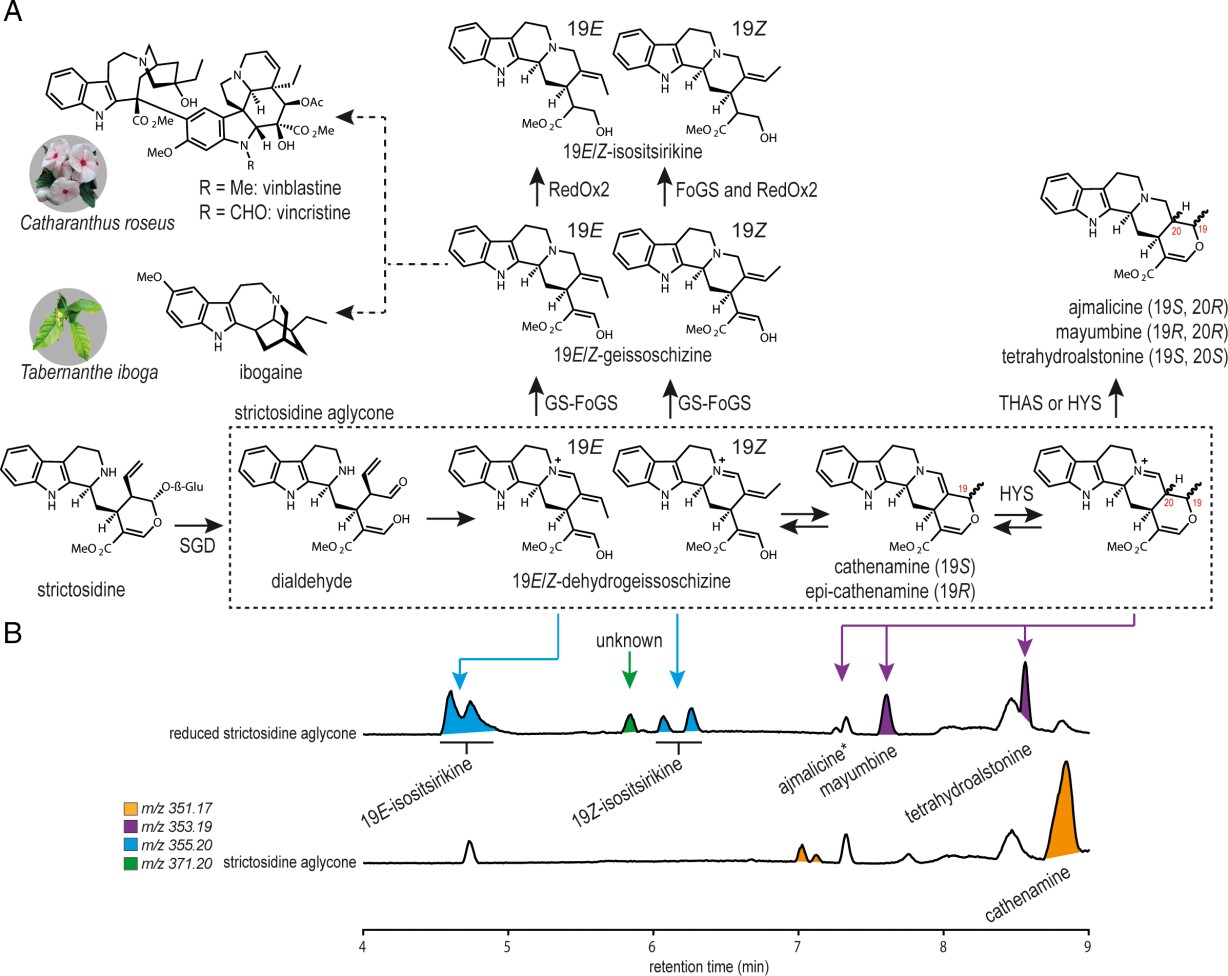
The strictosidine aglycone branch point. (*A*) The enzymes and intermediates of the strictosidine aglycone branch point in *Catharanthus roseus* and *Tabernanthe iboga* pathways. Major strictosidine aglycone isomers are shown within the dotted line. Solid arrows represent single enzymatic/chemical conversions and dotted arrows represent multi-enzyme steps. SGD, strictosidine β-glucosidase; GS, geissoschizine synthase; FoGS, facilitator of geissoschizine synthase; HYS, heteroyohimbine synthase; THAS, tetrahydroalstonine synthase. (*B*) LC–MS baseline peak chromatograms of strictosidine aglycone and the products of subsequent reduction by sodium borohydride. Peaks are highlighted based on *m/z* values. Ajmalicine is present at trace levels, but is obscured by a larger peak. Arrows are drawn to show the most likely strictosidine aglycone species that the subsequent reduction product corresponds to. Double peaks of 19*E*- and 19*Z*-isositsirikine correspond to 16*R* and 16*S* stereoisomers, previously described (17).

Monoterpene indole alkaloids are an important class of natural products produced by plant species in the order Gentianales, with over 3,000 structurally distinct molecules reported (1). The medicinal value of monoterpene indole alkaloids is exemplified by compounds such as vinblastine and vincristine (anticancer drugs) (2, 3), ibogaine (antiaddictive activity) (4, 5), ajmalicine (antihypertensive activity) (6), and quinine (antimalaria activity) (7). Given the structural complexity and pharmacological importance of these alkaloids, substantial efforts have been made to elucidate the pathways that encode the biosynthesis of these compounds. All monoterpene indole alkaloids are derived from strictosidine, an intermediate that is formed by enzyme-catalyzed condensation of tryptamine and the monoterpene secologanin (8). Strictosidine is deglycosylated by strictosidine β-glucosidase (SGD) to form a labile aglycone that can rearrange to form a variety of isomers (9, 10). These strictosidine aglycone isomers are reductively trapped by medium-chain dehydrogenases/reductases to generate the scaffolds for the major classes of monoterpene indole alkaloids (11–16) (Fig. 1). A particularly important strictosidine aglycone isomer is dehydrogeissoschizine, which is reduced by geissoschizine synthase (GS) to form 19*E*-geissoschizine (15), a precursor for strychnos, aspidosperma, and iboga type alkaloids, which include many medicinally important compounds including vinblastine and ibogaine.

Herein, we report facilitator of geissoschizine synthase (FoGS), a protein that aids in the formation of geissoschizine, likely through heterodimerization with GS. We identified orthologs of FoGS from the geissoschizine producers *C. roseus* and *T. iboga* and demonstrate activity for all orthologs in *Nicotiana benthamiana* and in vitro. Virus induced gene silencing (VIGS) demonstrates the clear involvement of FoGS in the geissoschizine-derived vinblastine biosynthetic pathway in *C. roseus*. FLIM-FRET and coimmunoprecipitation experiments showed protein–protein interactions between FoGS and GS, and suggest that the two proteins form a heterodimer. We show that FoGS modestly increases levels of the medicinal alkaloid catharanthine when expressed along with the previously known biosynthetic genes in *N. benthamiana* leaves. This work highlights how metabolic pathways utilize proteins that, when assayed alone appear nonessential, but when combined with the correct partner, provide enhanced efficiency or specificity to a given metabolic step. The availability of highly resolved datasets and high-throughput functional characterization screens will likely facilitate identification of more examples of such cryptic proteins.

## Results

### A Medium-chain Dehydrogenase/Reductase Facilitates *C. roseus* GS.

In *C. roseus*, strictosidine aglycone isomers are reductively trapped by one of the medium-chain dehydrogenases/reductases GS, HYS, or THAS (12, 13, 15). GS catalyzes conversion of strictosidine aglycone (reduction of dehydrogeissoschizine isomer) to 19*E-*geissoschizine, which is directed to strychnos/aspidosperma/iboga-type alkaloids. Alternatively, HYS and THAS reduce strictosidine aglycone (cathenamine isomer) to the heteroyohimbine-type alkaloids ajmalicine (HYS), tetrahydroalstonine (THAS or HYS), or mayumbine (HYS). Inspired by the chalcone isomerase-like proteins that enhance titers of flavonoid biosynthetic pathways (18–20), we speculated that an additional protein could assist the efficiency or specificity of the isomerization process involved in this step of the monoterpene indole alkaloid pathway.

Using a published *C. roseus* single-cell transcriptome dataset (21), we identified candidate genes based on co-clustering with SGD in epidermal cells (*SI Appendix*, Fig. S1). Candidates were screened in batches via agrobacterium-mediated expression in *N. benthamiana*. We tested all candidates in the presence of strictosidine and SGD to generate the strictosidine aglycone substrate. Additionally, we included the three major medium-chain dehydrogenases/reductases that act on strictosidine aglycone, GS1, HYS, or THAS1. This competitive assay would enable us to identify proteins that would alter the subsequent alkaloid product profiles: ajmalicine, mayumbine, and tetrahydroalstonine for HYS; tetrahydroalstonine for THAS1; and 19*E*-geissoschizine for GS1. In *N. benthamiana*, the enol moiety of geissoschizine stereoisomers (19*E* or 19*Z*) is further reduced by an endogenous reductase or reductases to form 19*E*- and 19*Z*-isositsirikine while retaining the original 19*E*/*Z*-stereochemistry set by GS (17) (*SI Appendix*, Fig. S2).

**Fig. 2.**
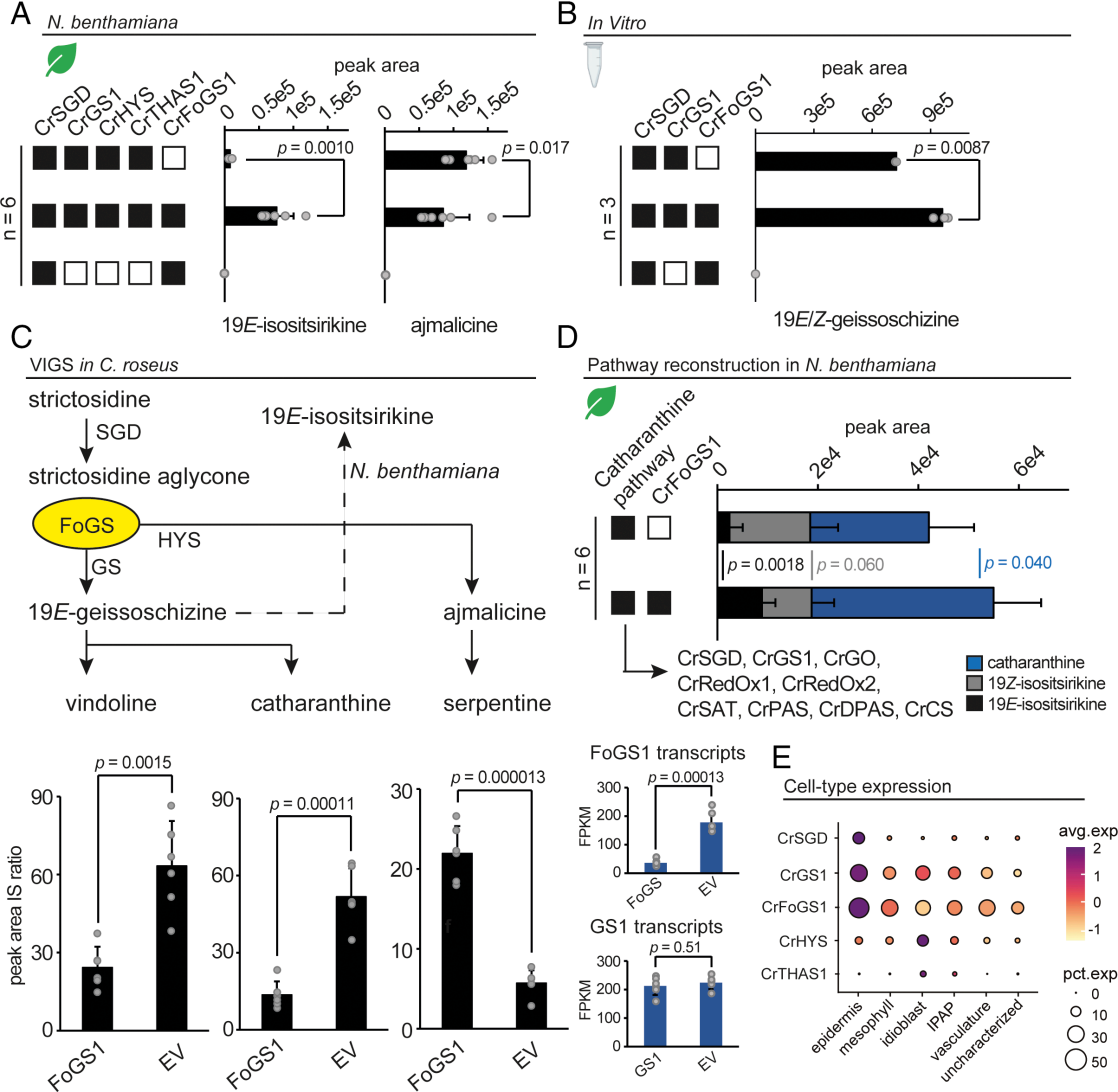
A medium-chain dehydrogenase/reductase facilitates the GS1 enzyme in *C. roseus*. For all bar graphs, values for individual replicates are shown as gray circles and averages as black bars. Error bars and *P*-values from Student’s *t* tests are shown. (*A*) Competitive SGD, GS1, HYS, THAS1 assays in *N. benthamiana* with and without FoGS1. Results for other candidates are shown in *SI Appendix*, Fig. S1. Each treatment is the average of six biological replicates. (*B*) In vitro characterization of GS1 and FoGS1. Combined peak areas of 19*E*- and 19*Z*-geissoschizine from three replicates are shown. (*C*) VIGS of FoGS1 in *C. roseus* leaves. A diagram highlighting the major enzymes, intermediates, and downstream products of the strictosidine aglycone branch point. Results from targeted metabolomics of vindoline, catharanthine, and serpentine are shown below the diagram alongside average FPKM values for FoGS1 and GS1. Average peak areas were determined from six biological replicates. (*D*) Reconstruction of the *C. roseus* 8-step biosynthetic pathway from strictosidine to catharanthine in *N. benthamiana* leaves. Each treatment is the average of six biological replicates. Blue corresponds to catharanthine, gray to 19*Z*-isositsirikine, and black to 19*E*-isositsirikine. Full dataset showing individual replicate values is shown in *SI Appendix*, Fig. S4. (*E*) Single cell transcriptomics for selected *C. roseus* genes. Although HYS is predominantly expressed in idioblast cells, HYS is expressed in both epidermal and idioblast cells under certain conditions (22). Single cell transcriptomics are represented by a dotplot where circle size corresponds to percent expression and circle color corresponds to average expression. SGD, strictosidine β-glucosidase; GS, geissoschizine synthase; FoGS1, facilitator of geissoschizine synthase; HYS, heteroyohimbine synthase; THAS, tetrahydroalstonine synthase; GO, geissoschizine oxidase; RedOx1, reductive oxidative enzyme 1; RedOx2, reductive oxidative enzyme 2; SAT, stemmadenine acetyltransferase; PAS, precondylocarpine acetate synthase; DPAS, dehydroprecondylocarpine synthase; CS, catharanthine synthase.

These *N. benthamiana* assays established that ajmalicine is the major product of HYS, a stark difference to the even distribution of ajmalicine, mayumbine, and tetrahydroalstonine that is produced by HYS in in vitro assays (13). We additionally noted that in this competitive assay, GS1 is outcompeted by HYS, reflected by the higher levels of ajmalicine compared to the 19*E*-geissoschizine-derived isositsirikine. Notably, one candidate increased flux through GS1 at the expense of HYS, as evident by an increase in 19*E*-isositsirikine and decrease in ajmalicine (Fig. 2*A* and *SI Appendix*, Fig. S1). This candidate, which was annotated as a medium-chain dehydrogenase/reductase, was unable to reduce strictosidine aglycone when expressed alone with SGD in the presence of strictosidine. We thus named this candidate facilitator of geissoschizine synthase (FoGS). FoGS was then assayed with each medium-chain dehydrogenase/reductase individually. Addition of FoGS to assays containing strictosidine, SGD, and GS1 resulted in a near significant increase (*P*-value of 0.058) in 19*E*-isositsirikine (*SI Appendix*, Fig. S3). Conversely, addition of FoGS to assays of strictosidine, SGD, and either HYS or THAS1 revealed no significant changes to the resulting product profiles (*SI Appendix*, Fig. S3). Since omission of THAS1 from competition assays had no effect on product profiles (*SI Appendix*, Fig. S3), we omitted THAS1 from subsequent experiments. This is congruent with single cell transcriptomics data that show THAS1 is not expressed in epidermal cells and is therefore spatially separated from SGD and GS1 (21, 22) (*SI Appendix*, Fig. S1).

To validate whether FoGS plays a role in 19*E*-geissoschizine synthesis in planta, we silenced FoGS in *C. roseus* leaves by VIGS. The silencing vector was designed to ensure no off-target effects. Silencing of FoGS resulted in an 80% decrease in transcript levels compared to empty vector controls without altering GS1 expression (FoGS and GS1 share 61% sequence identity). This was accompanied by a decrease in the 19*E*-geissoschizine-derived metabolites catharanthine (iboga type), vindoline (aspidosperma type), and an increase in the ajmalicine-derived metabolite serpentine (heteroyohimbine type) (Fig. 2*C*). Therefore, the VIGS data clearly support a role for FoGS in directing metabolic flux at the GS-HYS branch point that controls the switch between aspidosperma/iboga and heteroyohimbine-type alkaloids. The FoGS VIGS chemotype closely resembles what is observed when GS1 is subjected to silencing, for which a decrease in 19*E*-geissoschizine-derived metabolites and an increase in ajmalicine is also reported (23). These results suggest that FoGS enhances 19*E*-geissoschizine production at the strictosidine aglycone branch point.

### In Vitro Characterization of *Cr*FoGS.

To further support the role of FoGS, we expressed and purified SGD, GS1, and FoGS recombinantly for in vitro characterization. The in vitro enzyme assays were conducted by first deglycosylating strictosidine with SGD and allowing the aglycone to equilibrate before the addition of GS1 and FoGS. LC–MS analysis of equilibrated strictosidine aglycone revealed only one major peak with an *m/z* value of 351.17 (*SI Appendix*, Fig. S5). This product was purified by HPLC and NMR analysis indicated that this product was the strictosidine aglycone isomer cathenamine (*SI Appendix*, Figs. S6 and S7), consistent with previous reports (9, 10, 24). Quenching this aglycone reaction with sodium borohydride and comparison of the resulting product mixture with authentic standards, demonstrated that cathenamine can be chemically reduced to 19*E*-, 19*Z*-isositsirikine, mayumbine, tetrahydroalstonine, and ajmalicine (Fig. 1*B*). These results suggest that cathenamine is in equilibrium with the aglycone substrates (19*E*-, 19*Z*-dehydrogeissoschizine and tautomerized iminium forms of cathenamine and epi-cathenamine) that lead to the products of GS1 and HYS. Importantly, in vitro enzyme assays show that FoGS modestly increases GS1 catalyzed production of 19*E*- (major) and 19*Z*-geissoschizine (minor) (Fig. 2*B*). Interestingly, FoGS also displayed modest 19*Z*-isositsirikine synthase activity as evidenced by the reduction of 19*Z*-geissoschizine in coupled assays. This was confirmed with assays that incubated FoGS with synthetic 19*E*- and 19*Z*-geissoschizine as substrates (*SI Appendix*, Fig. S8). In extended assays, FoGS reduced strictosidine aglycone to form two unknown products with *m/z* values of 353.19 and 355.20, though the low titers of these products prevented isolation and structural characterization. These products were not observed in *N. benthamiana* assays (*SI Appendix*, Fig. S9). Therefore, FoGS retains the catalytic capacity for reduction, though this activity does not appear to be utilized in geissoschizine production.

### *T. iboga* FoGS Orthologs Provide Added *E/Z*-stereochemical Control.

**Fig. 3.**
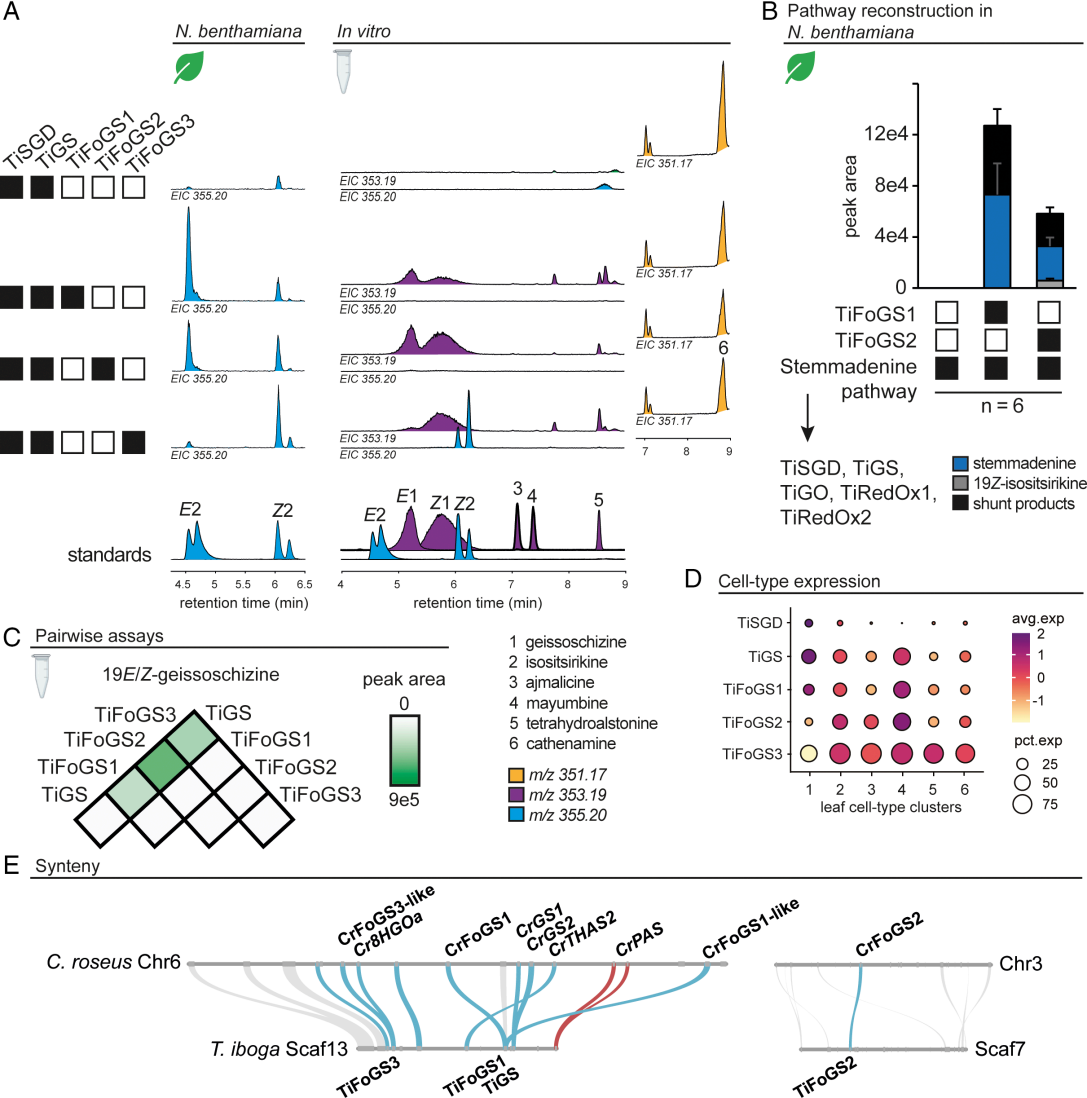
FoGS orthologs from *T. iboga*. (*A*) Characterization of *Ti*FoGS1, *Ti*FoGS2, and *Ti*FoGS3 with *Ti*SGD and TiGS in *N. benthamiana* and in vitro. Extracted ion chromatograms (EIC) are shown for *m/z* values of 353.19 (purple), 355.20 (cyan), and 351.17 (orange). Standards for 19*E*/*Z*-geissoschizine (1), 19*E*/*Z*-isositsirikine (2), ajmalicine (3), mayumbine (4), and tetrahydroalstonine (5) are shown below. (*B*) Reconstruction of the *T. iboga* 4-step *biosynthetic* pathway from strictosidine to stemmadenine in *N. benthamiana* leaves. Each treatment is the average of six biological replicates. Blue corresponds to stemmadenine, gray to 19*Z*-isositsirikine, and black to shunt products. Shunt products are formed endogenously in *N. benthamiana* from stemmadenine. Full dataset showing individual replicate values is shown in *SI Appendix*, Fig. S11. (*C*) In vitro assays of all pairwise combinations of *Ti*GS, *Ti*FoGS1, *Ti*FoGS2, and *Ti*FoGS3. The average total 19*E*/*Z*-geissoschizine peak area is shown as a color scale based on three replicates. (*D*) Single cell transcriptomics for select *T. iboga* genes, represented by a dotplot where circle size corresponds to percent expression and circle color corresponds to average expression. (*E*) Microsynteny comparison highlights *C. roseus* and *T. iboga* genomic regions containing FoGS genes. Blue lines indicate synteny between medium-chain dehydrogenases/reductases, red for flavin-containing berberine bridge-like proteins (PAS), and gray for other. SGD, strictosidine β-glucosidase; GS, geissoschizine synthase; FoGS, facilitator of geissoschizine synthase; THAS, tetrahydroalstonine synthase; GO, geissoschizine oxidase; RedOx1, reductive oxidative enzyme 1; RedOx2, reductive oxidative enzyme 2; PAS, precondylocarpine acetate synthase; 8HGO, 8-hyrdroxygeraniol oxidoreductase.

We next asked whether FoGS orthologs are utilized in other geissoschizine producing species. *T. iboga*, which produces the antiaddiction agent ibogaine and is phylogenetically related to *C. roseus*, was targeted for analysis. GS from *T. iboga* is a catalytically inefficient enzyme that produces 19*Z*-geissoschizine as a major product, even though the downstream enzyme, geissoschizine oxidase (GO), only turns over 19*E*-geissoschizine (17). In our hands, *Ti*GS showed trace 19*Z*-GS activity in *N. benthamiana* and no detected activity in vitro (Fig. 3*A*). We suspected that a FoGS ortholog may be required for production of 19*E*-geissoschizine by *Ti*GS. *Ti*FoGS candidates were selected based on sequence similarity with *Cr*FoGS1 via BLAST analysis of a published *T. iboga* transcriptome (25). Candidates were initially screened via agrobacterium-mediated expression in *N. benthamiana* with *Ti*SGD and *Ti*GS and strictosidine as a starting substrate. Again, since *N. benthamiana* reduces *E/Z*-geissoschizine, levels of the reduced product, *E/Z*-isositsirikine, were measured in the assays. Three candidates, *Ti*FoGS1, *Ti*FoGS2, and *Ti*FoGS3, all substantially increased the levels of geissoschizine produced by *Ti*GS, with varying degrees of 19*E*/*Z* specificity (Fig. 3*A*). Addition of *Ti*FoGS1 to the assay enabled formation of 19*E*- (major) and 19*Z*-geissoschizine (minor) by *Ti*GS, similar to *Cr*FoGS, thereby explaining how 19*E*-geissoschizine, required for ibogaine biosynthesis, is produced in this plant. *Ti*FoGS2 had a similar effect as *Ti*FoGS1 but resulted in a mixture of 19*E*- and 19*Z*-geissoschizine in an approximately 1:1 ratio. Interestingly, assays of *Ti*FoGS3 with *Ti*GS primarily produced the 19*Z*-geissoschizine isomer. These results were reproduced in vitro with purified protein (Fig. 3*A*). When expressed alone with *Ti*SGD, *Ti*FoGS1, *Ti*FoGS2, and *Ti*FoGS3 all showed low levels of GS-activity in *N. benthamiana* but not in vitro (*SI Appendix*, Fig. S10). However, in vitro assays strongly suggest that *Ti*GS is integral to geissoschizine production, since no pairwise combinations of *Ti*FoGS1, *Ti*FoGS2, or *Ti*FoGS3 produced geissoschizine in vitro (Fig. 3*C*). *Ti*FoGS orthologs also converted strictosidine aglycone to the same unknown product (*m/z* 353.19) observed with *Cr*FoGS1 (*SI Appendix*, Fig. S9). Only *Ti*FoGS3 could reduce 19*Z*-geissoschizine to 19*Z*-isositsirikine (Fig. 3*A*). Using a deposited single-cell transcriptome from *T. iboga* leaves (*SI Appendix*, *Supplemental Methods*), we observed that *Ti*FoGS1 is most clearly clustered with *Ti*SGD and *Ti*GS (Fig. 3*D*). We therefore propose that *Ti*FoGS1 is the major contributor to GS-catalyzed production of 19*E*-geissoschizine in *T. iboga*.

### FoGS Is Located in a Biosynthetic Gene Cluster with GS.

Synteny analysis using published *C. roseus* (21) and *T. iboga* genomes (*SI Appendix*, *Supplemental Methods*) revealed that *Cr*FoGS, *Ti*FoGS1, and *Ti*FoGS3 are located in a previously noted genomic region containing GS1, GS2, THAS1, as well as two additional monoterpene indole alkaloid genes (8HGOa and PAS) (26) (Fig. 3*E*). *Ti*FoGS1 and *Ti*FoGS3 are syntenic to *Cr*FoGS and *Cr*8HGOa, respectively, alongside two uncharacterized medium-chain dehydrogenases/reductases. *Ti*FoGS2 is located outside the gene cluster and is syntenic to an uncharacterized *C. roseus* medium-chain dehydrogenase/reductase. Single-cell transcriptomics of the newly identified *C. roseus* FoGS syntenologs reveal that only *Cr*FoGS1 is coexpressed in epidermal cells with *Cr*GS1 (*SI Appendix*, Fig. S12). FoGS-activity was detected for *Cr*FoGS2 and *Cr*8HGOa but not for *Cr*FoGS3-like when assayed in *N. benthamiana* (*SI Appendix*, Fig. S12). However, *Cr*FoGS2 and *Cr*8HGOa did not provide the observed 19*E*/*Z*-stereochemical control exhibited by the *T. iboga* counterparts. The syntenic *Cr*GS and *Ti*GS share a conserved catalytic motif. This motif has been reported in iminium reducing medium-chain dehydrogenases/reductases from monoterpene indole alkaloid biosynthesis, including other characterized GS enzymes (27, 28). Conversely, all reported FoGS proteins retain the canonical medium-chain dehydrogenase/reductase catalytic motif (*SI Appendix*, Fig. S13). Phylogenetic analysis demonstrates that GS, FoGS1, 2, and FoGS3 orthologs clade separately alongside medium-chain dehydrogenase/reductase family members with reported roles in biosynthesis of these alkaloids (*SI Appendix*, Fig. S13).

### Protein–Protein Interactions between *C. roseus* FoGS1 and GS1.

We hypothesized that protein–protein interactions between FoGS and GS contribute to the observed role of FoGS. We first assessed the subcellular localization of the *C. roseus* strictosidine aglycone branch point enzymes, SGD, GS1, FoGS1, and HYS, by transiently expressing GFP/RFP fusion constructs in *N. benthamiana* leaves (*SI Appendix*, Figs. S14–S17). *Cr*SGD and *Cr*HYS demonstrated similar subcellular localization patterns as previously reported (13, 24, 29). *Cr*SGD formed dense *fusiform* nuclear structures that were disrupted by C-terminal tagging (*SI Appendix*, Fig. S15), and *Cr*HYS displayed nucleocytosolic localization. *Cr*GS1 showed nucleocytosolic localization with a strong nuclear signal, while *Cr*FoGS1 displayed nucleocytosolic localization with a weak nuclear signal. Unexpectedly, when coexpressed with *Cr*FoGS1, the subcellular localization of *Cr*GS1 was modulated, appearing predominantly excluded from the nucleus, thereby matching the localization of *Cr*FoGS (*SI Appendix*, Fig. S16). *T. iboga* SGD, GS, and FoGS1 displayed near identical patterns of subcellular localization as the *C. roseus* orthologs when expressed in *N. benthamiana* (*SI Appendix*, Fig. S18). Importantly, we observed the same exclusion of GS from the nucleus when coexpressed with FoGS1 (*SI Appendix*, Figs. S19–S22).

**Fig. 4.**
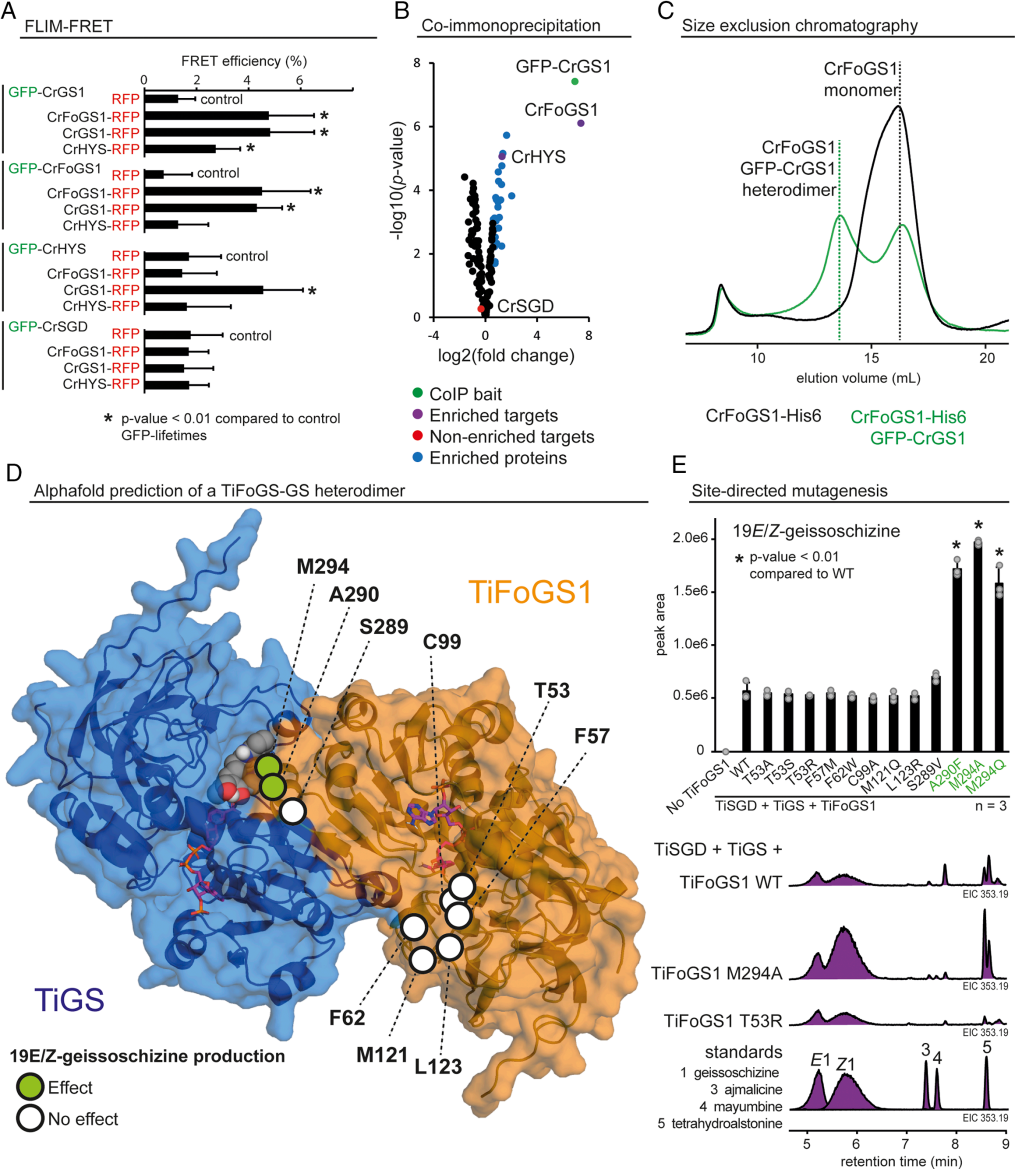
Protein–protein interactions between FoGS1 and GS. (*A*) Pairwise FLIM-FRET assays between *Cr*SGD, *Cr*GS1, *Cr*FoGS1, and *Cr*HYS expressed in *N. benthamiana* leaves. FRET efficiencies are shown as an average of six measurements from three biological replicates. Errors bars are shown. Treatments which had significantly different GFP fluorescence lifetimes compared to free-RFP negative controls (*P*-values from Student’s *t* tests < 0.01) are marked with an asterisk. GFP fluorescence lifetimes are shown in *SI Appendix*, Fig. S23. (*B*) Volcano plot showing the enrichment of proteins between GFP-*Cr*GS1 Co-IPs and free-GFP negative controls. The results were determined from three biological replicates. GFP-*Cr*GS1 (bait) is shown in green, enriched proteins in blue, enriched proteins of interest in purple, and nonenriched proteins of interest in red. (*C*) Size exclusion chromatography of the purified *C. roseus* FoGS1–GS1 complex. Chromatogram for His^6^-*Cr*FoGS1 expressed alone is shown in black and His^6^-*Cr*FoGS1 coexpressed with GFP-*Cr*GS1 is shown in green. SDS-PAGE and Western blots of collected fractions and the calibration curve used to calculate molecular weight are shown in *SI Appendix*, Fig. S25. (*D*) Alphafold3 prediction (ipTM = 0.94; pTM = 0.93) of the *T. iboga* FoGS1–GS heterodimer. *Ti*GS is shown in blue, *Ti*FoGS1 in orange, NADPH in magenta, and docked 19*E*-dehydrogeissoschizine as gray spheres. The location of amino acids from site-directed mutagenesis of *Ti*FoGS1 and the corresponding effect on geissoschizine formation (green for effect, white for no effect) are mapped onto the model. (*E*) In vitro GS-coupled assays of *Ti*FoGS1 variants. Bar graphs of combined 19*E*- and 19*Z*-geissoschizine peak area are shown for all *Ti*FoGS1 variants. Values for individual replicates are shown as gray circles and averages as black bars. Significant changes (*P*-values from Student’s *t* tests < 0.01) are marked with an asterisk. Select EICs are shown for *m/z 353.19* in purple. Standards for 19*E*/*Z*-geissoschizine (1), ajmalicine (3), mayumbine (4), and tetrahydroalstonine (5) are shown below. Full results from site-directed mutagenesis are shown in *SI Appendix*, Fig. S26. SGD, strictosidine β-glucosidase; GS, geissoschizine synthase; FoGS, facilitator of geissoschizine synthase; HYS, heteroyohimbine synthase.

Although subcellular expression patterns of overexpressed proteins must be interpreted with caution, these data suggest that FoGS and GS interact. We used fluorescence lifetime imaging microscopy (FLIM) to detect Förster resonance energy transfer (FRET) between GFP and RFP protein fusions transiently expressed in *N. benthamiana* leaves. In pairwise FLIM-FRET assays between *C. roseus* GS1, FoGS1, HYS, and SGD, FRET was only observed for *Cr*GS-FoGS1 (4.8 to 5.5% FRET) and *Cr*GS-HYS (2.7 to 4.5% FRET) (Fig. 4*A* and *SI Appendix*, Fig. S23). We also note the unusual behavior of C-terminally tagged SGD, for which FRET signal was measured in all assays including a free-GFP negative control (*SI Appendix*, Fig. S23). To validate the results of the FRET experiments, we performed coimmunoprecipitation from *N. benthamiana* leaves transiently expressing *C. roseus* SGD, GS1, FoGS1, and HYS using GFP-*Cr*GS1 as bait. Co-immunoprecipitated proteins were analyzed by SDS-PAGE and proteomic analysis. Proteomics showed that only *Cr*FoGS1 was strongly enriched with GFP-*Cr*GS1 in coimmunoprecipitation assays, while *Cr*HYS showed modest enrichment, and *Cr*SGD was not enriched at all (Fig. 4*B*). Congruently, coimmunoprecipitation experiments using GFP-*Cr*SGD as bait demonstrated a lack of enrichment for *Cr*GS1, *Cr*FoGS1, and *Cr*HYS (*SI Appendix*, Fig. S24). We also observed the appearance of a strong protein band in the SDS-PAGE of GFP-*Cr*GS1 coprecipitated proteins. This band had the molecular weight of a medium-chain dehydrogenase/reductase and likely corresponds to *Cr*FoGS1 based on the proteomics data (*SI Appendix*, Fig. S24). Collectively, these results suggest that *Cr*FoGS1 and *Cr*GS1 form a stable complex.

### Heterodimerization of FoGS and GS.

To investigate the molecular arrangement of the FoGS1–GS interaction, we purified the complex from *N. benthamiana* leaves coexpressing His^6^-*Cr*FoGS1 and GFP-*Cr*GS1. His^6^-*Cr*FoGS1 and interacting GFP-*Cr*GS1 were first captured using Ni-NTA affinity chromatography, then subsequently separated by molecular weight using size exclusion chromatography. When His^6^-*Cr*FoGS1 was expressed in the absence of GFP-*Cr*GS1, a single peak was observed during size exclusion chromatography with an elution volume corresponding to monomeric *Cr*FoGS1 (Fig. 4*C*). In contrast, when His^6^-*Cr*FoGS1 was coexpressed with GFP-*Cr*GS1, an additional higher molecular weight peak was observed (Fig. 4*C*). SDS-PAGE and Western blots confirmed that the additional peak contains both *Cr*FoGS1 and GFP-*Cr*GS1 (*SI Appendix*, Fig. S25). Importantly, the elution volume of this peak corresponds to that of a GFP-*Cr*GS1-*Cr*FoGS1 heterodimer. We observed the same peak distribution during size exclusion chromatography of purified *T. iboga* His^6^-FoGS1 and YFP-GS, demonstrating that FoGS1–GS heterodimerization is conserved in *C. roseus* and *T. iboga* (*SI Appendix*, Fig. S25). Modeling with Alphafold3 suggests that the FoGS1–GS heterodimer possesses the same quaternary structure as the empirically determined *Cr*GS1 homodimer (Fig. 4*D*) (27). This mode of dimerization is highly conserved in other medium-chain dehydrogenases/reductases, including examples from the monoterpene indole alkaloid pathway [(13, 27, 30), reviewed in ref. 28]. The resulting quaternary organization produces two binding pockets that are predominantly located within each protomer but have a distinct contribution of a short α-helix from the neighboring protomer. In this manner, the heterodimerization of FoGS and GS would result in two binding pockets: a GS binding pocket with FoGS contribution, and a FoGS binding pocket with GS contribution.

To test the predicted model, we made single amino acid substitutions to the canonical medium-chain dehydrogenase/reductase binding pocket of *Ti*FoGS1 and tested the effect in combination with wild-type *Ti*GS in vitro. *T. iboga* enzymes were used because of the clear qualitative effect of *Ti*FoGS1 on *Ti*GS. Binding pocket residues were selected based on an Alphafold3 model of a *Ti*FoGS1 homodimer (*SI Appendix*, Fig. S26). Only two positions (M294 and A290) resulted in a change in the relative levels of 19*E*- and 19*Z*-geissoschizine formation. The M294A, M294Q, and A290F *Ti*FoGS1 variants increased both the level of total geissoschizine and the ratio of 19*Z*- to 19*E*-geissoschizine (Fig. 4*E* and *SI Appendix*, Fig. S26). Mapping the tested *Ti*FoGS1 binding pocket residues on the *Ti*FoGS1-*Ti*GS heterodimer model strongly suggests that M294 and A290 interact with *Ti*GS and are not part of the *Ti*FoGS1 binding pocket (Fig. 4*D*). Similarly, the S289V *Ti*FoGS1 variant also interacts with *Ti*GS in the heterodimer model and resulted in an apparent, but statistically insignificant (*P* = 0.09), increase in total geissoschizine. All other tested positions were predicted to be located in the *Ti*FoGS1 binding pocket and did not alter 19*E*/*Z*-geissoschizine formation (Fig. 4*E* and *SI Appendix*, Fig. S26). Notably, bulky substitutions intended to disrupt the *Ti*FoGS1 active site (T53R and L123R) had no noticeable effect on 19*E*/*Z*-geissoschizine formation, but did result in the decrease of FoGS-specific products (*m/z* value of 353.19). Additionally, C99A and T52S *Ti*FoGS1 variants increased the formation of FoGS-specific products without altering 19*E*/*Z*-geissoschizine formation. The effect on geissoschizine formation resulting from mutation of *Ti*FoGS1 residues that are proximal to the *Ti*GS binding pocket, and the lack of effect resulting from mutation of *Ti*FoGS1 binding pocket residues, are suggestive that dehydrogeissoschizine reduction is mediated by the GS-subunit of the GS–FoGS complex. Although our modeling suggests that *Ti*FoGS1 directly contributes to the GS binding pocket, given the lack of an empirical heterodimer structure we cannot exclude that other cross-subunit functional effects may be responsible for the combined activity of FoGS and GS.

### Pathway Reconstruction in *N. benthamiana*.

We next tested whether FoGS could be leveraged to improve the heterologous pathway reconstruction in *N. benthamiana* leaves. To do this, we coexpressed the genes from *C. roseus* responsible for converting strictosidine to catharanthine in *N. benthamiana* with and without *Cr*FoGS1. The addition of *Cr*FoGS1 resulted in a modest increase of catharanthine and 19*E*-isositsirikine peak areas (Fig. 2*D*). Additionally, the same experiment was conducted for the *T. iboga* genes responsible for converting strictosidine to stemmadenine using *Ti*FoGS1 and *Ti*FoGS2. In this case, *Ti*FoGS1 resulted in the greatest increase in stemmadenine peak area compared to *Ti*FoGS2, for which 19*Z*-isositsirikine was also observed (Fig. 3*B*). This result is consistent with the 19*E*- and 19*Z*-geissoschizine stereospecificity imparted by *Ti*FoGS2 and *Ti*GS. Collectively, these results reinforce the biosynthetic role of FoGS and demonstrate its potential to improve, albeit modestly, titers of valuable monoterpene indole alkaloids in heterologous expression systems.

## Discussion

Here, we report FoGS, a protein that enhances the efficiency and specificity of the central monoterpene indole alkaloid biosynthetic enzyme GS. Orthologs of FoGS were identified in two related plants, *C. roseus* and *T. iboga*, both of which produce geissoschizine. Inclusion of the *Cr*FoGS1 protein only modestly impacted the yields of the *C. roseus* GS1 ortholog in a stand-alone assay. However, when *Cr*FoGS1 was assayed in a competition assay in which strictosidine aglycone was incubated with multiple enzymes that compete for the strictosidine aglycone substrate (*Cr*HYS and *Cr*THAS1), higher relative levels of the *Cr*GS1 product were observed. Moreover, silencing of *Cr*FoGS1 in *C. roseus* leaves showed that absence of this protein significantly lowers the levels of downstream geissoschizine-derived metabolites in planta. The *T. iboga* GS ortholog absolutely requires FoGS1 to catalyze the formation of 19*E*-geissoschizine. Interestingly, we identified an additional *T. iboga* ortholog, *Ti*FoGS3, that when combined with *Ti*GS, imparts 19*Z*-stereochemical control over the reduction of dehydrogeissoschizine. A biological role for *Ti*FoGS3 in geissoschizine biosynthesis is unlikely since *Ti*GS and *Ti*FoGS3 are not coexpressed in the same cell type. However, the stereochemical control imparted by *Ti*GS, *Ti*FoGS1, and *Ti*FoGS3 provides an intriguing opportunity to access 19*E*- and 19*Z*-geissoschizine for biotechnological applications. Furthermore, this constitutes an unusual phenomenon in which the stereochemical outcome of an enzyme-catalyzed reaction can be modulated by partner proteins.

FLIM-FRET and coimmunoprecipitation experiments convincingly showed that *Cr*FoGS1 and *Cr*GS1 interact. Additionally, heterologously expressed *C. roseus* and *T. iboga* FoGS1–GS heterodimers could be purified from *N. benthamiana* leaves. The quaternary organization of FoGS and GS in the heterodimeric structure that is predicted by Alphafold3 suggests that FoGS comprises part of the binding pocket of GS, and vice versa. This predicted mode of heterodimerization is congruent with the highly conserved mode of homodimerization within the medium-chain dehydrogenase/reductase family (28). Site-directed mutagenesis of *Ti*FoGS1 suggests that, out of all tested residues, only those at the predicted FoGS-GS interface, near the GS binding pocket, influence geissoschizine formation. Based on the crystal structure of GS, the Alphafold3 heterodimer model, and these mutagenesis studies, we hypothesize that upon heterodimerization with GS, FoGS contributes amino acid side chains (M294 and A290) that remodel the GS binding pocket. However, empirical structural data will be needed to test this hypothesis. Additionally, careful characterization of these enzymes in the native environment will be required to validate this hypothesis of FoGS and GS orchestration.

The enzyme GS has been proposed to be the sole protein responsible for 19*E*-geissoschizine formation. The work reported here reveals that a partner protein, FoGS, substantially contributes to the efficiency and stereoselectivity of geissoschizine synthesis. This work thus highlights the potential for missing proteins in metabolic pathways. As the biosynthetic routes to many of these valuable compounds are elucidated (9, 16, 23, 31–34), additional factors that contribute to alkaloid biosynthesis may be discovered. Indeed, numerous proteins with cryptic functions have been reported to contribute to the biosynthesis of other plant natural products. In flavonoid biosynthesis, chalcone isomerase-like (CHIL) rectifies derailment caused by lactonization during the chalcone synthase (CHS) catalyzed formation of naringenin chalcone (18–20). Facilitator of taxane oxidation (FoTO1) was shown to eliminate shunt products not conducive to downstream biosynthesis during the oxidation of taxadiene from taxol biosynthesis in yew (35). Both CHIL and FoTO1 were shown to interact with the corresponding partner enzyme (19, 20, 35–37). *O*-methyltransferase heterodimerization was shown to set substrate specificity in noscapine biosynthesis from opium poppy (38). Contributions from metabolite-binding proteins have also been reported, as exemplified by the alkaloid-binding major latex proteins (MLPs) from opium poppy (39) and the flavonoid-binding pathogenesis-related 10 proteins (PR10s; analogous to MLP) from strawberry (40). Recently, a PR10 from *Marchantia polymorpha* was shown to protect naringenin chalcone from spontaneous cyclization in flavonoid biosynthesis (41). Proteins in lignin (42) and steroidal alkaloid biosynthesis (43) are hypothesized to enhance product titers by acting as a scaffold for the biosynthetic enzymes. Core eudicots synthesize indole by utilizing an inactive paralog of the tryptophan synthase β subunit (TSB-like), which in turn disrupts the canonical tryptophan synthase multimer (44). Additionally, in *C. roseus*, an alternate and inactive splice variant of SGD was shown to inhibit regular SGD function (24).

These examples point to an arising paradigm in natural product biosynthesis, where the more cryptic roles of proteins in biosynthetic pathways are being elucidated. The identification of FoGS highlights the involvement of proteins in metabolic pathways that, when assayed in isolation appear nonessential, but when assayed in combination with other pathway enzymes, increase the efficiency or specificity of a given metabolic step. The modest effect of FoGS in heterologous pathway reconstruction and its control over product stereochemistry could be applied to leverage known monoterpene indole alkaloid biosynthetic pathways for biotechnological applications.

## Materials and Methods

Additional materials and methods are described in *SI Appendix*.

### Candidate Selection.

Candidates were selected using a previously published *C. roseus* leaf single cell RNA-Seq dataset (21) that was reprocessed (alignment, clustering, and cell annotation) as previously reported. Transcripts annotated as major latex proteins, pathogenesis-related 10 proteins, alpha/beta-hydrolases, or medium-chain dehydrogenases/reductases (including cinnamyl-aldehyde/alcohol dehydrogenases) were targeted. Major latex proteins and pathogenesis-related 10 proteins were selected based on reported alkaloid-binding activities (39, 40), and medium-chain dehydrogenases/reductases were selected because of the roles of GS, HYS, THAS, DCS, and YOS in reducing strictosidine aglycone (11–14, 16). Alpha/beta-hydrolases were selected based on reported cyclase activities in monoterpene indole alkaloid (MIA) biosynthesis (32). An initial list of 222 candidates was filtered based on Pearson correlation with SGD cell type expression, and the level of expression in epidermal clusters. Only candidates with no reported MIA biosynthetic roles that showed a Pearson correlation with SGD expression greater than 0.5 and exhibited expression in the epidermal cell cluster greater than 1 (average normalized counts per 10,000) were selected for screening. This yielded 6 major latex proteins/pathogenesis-related 10 proteins, 3 alpha/beta-hydrolases, and 3 medium-chain dehydrogenases/reductases (*SI Appendix*, Fig. S1).

### Gene Screening and Pathway Reconstruction in *N. benthamiana* Leaves.

Two-days post agrobacterium infiltration, *N. benthamiana* leaves transiently expressing the genes pertaining to the given experiment were infiltrated with 150 μM substrate in 50 mM HEPES pH 7.5 into the abaxial side of leaves. Leaf discs were excised 3-d post substrate infiltration and metabolites were extracted in 400 μL methanol with gentle shaking. The sample was filtered through 0.45 μm low-binding hydrophilic polytetrafluoroethylene spin-filter plates (*Millipore*) and directly analyzed by liquid-chromatography–mass spectrometry (LC–MS). Analytes were verified with authentic standards based on retention time and MS/MS data (*SI Appendix*, Fig. S27). *N. benthamiana* assays were performed with four to six biological replicates, treating individual leaves from different plants as biological replicates.

### In Vitro Enzyme Assays.

Coupled SGD-GS enzyme assays were performed stepwise by first deglycosylating strictosidine with SGD then adding GS with and without FoGS. In the first step, 200 μM of strictosidine was deglycosylated by 20 nM SGD in 50 mM HEPES pH 7.5 and given 2 h to equilibrate at 30 °C. We identified cathenamine as the major product of this reaction (*SI Appendix*, Figs. S5–S7). Cathenamine reaction mixtures were then used as substrate for 50 μL enzyme assays of 0.25 μM GS, 1 μM FoGS, 1 mM NADPH, 0.1 mg/mL BSA, and 50 mM HEPES pH 7.5 at a final cathenamine concentration of 100 μM (assuming 100% conversion from strictosidine). Mixtures of GS and FoGS were pre-equilibrated for 30 min at 30 °C before the addition of cathenamine. *C. roseus* assays were run for 10 min while *T. iboga* assays were run for 1 h, both at 30 °C. Extended assays of *C. roseus* FoGS1 in the absence of GS were run for 1 h. *C. roseus* FoGS1 19*E*- and 19*Z*-geissoschizine assays were run for 1 h using the same conditions with 50 μM geissoschizine. All assays were quenched with 4-volumes of 100% methanol, vortexed, then centrifuged for 30 min at 4 °C to pellet proteins. Samples were filtered through 0.45 μm low-binding hydrophilic polytetrafluoroethylene spin-filter plates (*Millipore*) and analyzed using the same LC–MS method as for *N. benthamiana* assays. In vitro enzyme assays were performed in triplicate and analytes were verified with authentic standards based on retention time and MS/MS data (*SI Appendix*, Fig. S27).

### Fluorescence Lifetime Imaging Microscopy–Förster Resonance Energy Transfer (FLIM-FRET).

FLIM-FRET was performed as previously described (45). Briefly, eGFP tagged (N-terminal) and mRFP1 tagged (C-terminal) genes of interest were overexpressed in *N. benthamiana* leaves. Leaf discs were excised 3-d post agrobacterium infiltration and mounted in water. FLIM imaging was performed using Time-Correlated Single-Photon Counting (TCSPC) as implemented on a Stellaris 8 FALCON STED (*Leica*) equipped with a HC PL APO 40x/1.25 glycerol immersion objective (Leica). GFP was excited using a White Light Laser (WLL) (Leica) at a wavelength of 489 nm with 80 MHz pulse frequency. GFP emission between 495 and 583 nm was recorded using a HyD-X detector until reaching a photon/pixel count of 1,000. An additional channel was used to image RFP fluorescence using an excitation wavelength of 590 nm and emissions between 597 and 640 nm were recorded using a HyD-S detector. GFP fluorescence lifetimes were determined from phasor plots (46), as implemented in Leica Application Suite X v.4.8.1. FRET efficiencies were calculated with Eq. **1** (47) from six biological replicates. *τ*_unquenched_ corresponds to the fluorescence lifetime of eGFP alone, and *τ*_quenched_ corresponds to the fluorescence lifetime of eGFP in the presence of the acceptor fluorophore mRFP1.

[1] $$\mathrm{FRET}\;\mathrm{efficiency}=1-\tau_{\text{quenched}}/\tau_{\text{unquenched}}.$$

### Co-immunoprecipitation.

Co-immunoprecipitation of GFP tagged (N-terminal) *Cr*GS1 and *Cr*SGD with the strictosidine aglycone branch point enzymes (*Cr*SGD, *Cr*GS1, *Cr*FoGS1, and *Cr*HYS) were performed using a µMACS^TM^ GFP isolation kit (Miltenyi Biotech) following the manufacturer’s instructions. Briefly, GFP tagged (N-terminal) *Cr*GS1, *Cr*SGD, and free-GFP were overexpressed in *N. benthamiana* leaves with *Cr*SGD, *Cr*GS1, *Cr*FoGS1, and *Cr*HYS. Leaves were harvested 3-d post agrobacterium infiltration, flash frozen in liquid nitrogen, and grounded by mortar and pestle. Proteins were extracted from 200 mg of leaf tissue in 1 mL of lysis buffer (150 mM NaCl, 1% Ecosurf, 50 mM Tris-Cl pH 8) with an EDTA-free protease inhibitor cocktail for 1 h at 4 °C with gentle shaking. Cell debris was removed by centrifugation at 14,000 rpm. Then, 50 µL of anti-GFP magnetic beads were added to the supernatant and incubated for 30 min at 4 °C with gentle shaking. Samples were loaded onto µ Columns (*Miltenyi Biotech*) equilibrated in lysis buffer. Samples were washed four times with 200 µL wash buffer 1 and once with 100 µL wash buffer 2. Proteins were incubated for 5 min with 20 µL of hot SDS-elution buffer then eluted with 50 µL of hot SDS-elution buffer. Samples were subjected to SDS-PAGE and GFP or GFP-tagged proteins were detected by Western Blotting using a GFP polyclonal antibody-horseradish peroxidase conjugate (*Invitrogen*) at a 1:1,000 dilution followed by detection using Clarity Western ECL Substrate (*Bio-Rad*). Proteomics analysis was performed at the Proteomics core Facility, EMBL (Heidelberg, Germany). Co-immunoprecipitations were done in triplicate.

### Purification of the GS–FoGS1 Complex.

Open reading frames from *Cr*FoGS1 and *Ti*FoGS1 pOPINF constructs, including N-terminal His^6^ tags, were PCR amplified and assembled into pCambia vectors by In-Fusion. His^6^-FoGS was coexpressed with GFP/YFP tagged (N-terminal) GS in *N. benthamiana* leaves. For each treatment, 3 leaves from 10 plants were infiltrated. Three-days post agrobacterium infiltration, leaf tissue was flash frozen in liquid nitrogen and grounded by mortar and pestle. Pulverized plant tissue was incubated with 40 mL of buffer A1 supplemented with an EDTA-free protease inhibitor cocktail for 2 h at 4 °C with gentle shaking. Cell debris was removed by centrifugation at 35,000×*g* at 4 °C for 30 min, then filtered using Ministart^®^ NML Plus high-capacity syringe filters (*Sartorius*). Recombinantly expressed proteins were purified using an AKTA pure FPLC (*Cytiva*). Cleared lysate was loaded onto a His-Trap HP 5 mL column (*Cytiva*) at 2 mL/min equilibrated in buffer A1 and washed with 20 column volumes of buffer A1. Isocratic elution of His^6^-tagged proteins was performed with 100 % buffer B1 and 1.5 mL fractions were collected. Fractions corresponding to a single peak detected by UV at 280 nm were pooled. Size exclusion chromatography was then used to determine the molecular weight of proteins in the pooled fractions. Protein (400 µL) was injected onto a Superdex 200 10/300 GL size exclusion column (*Cytiva*) equilibrated in buffer A4 with a constant flow of 0.5 mL/min collecting 1 mL fractions. Eluting proteins were detected by UV at 280 nm. Fractions from size exclusion chromatography were analyzed by SDS-PAGE and GFP-tagged *Cr*GS1 detected by Western Blotting, as for coimmunoprecipitations. Molecular weights were calculated using a 4-point standard curve consisting of Catalase (240 kDa), monomeric and dimeric BSA (66 and 132 kDa), and Carbonic Anhydrase (30 kDa). All elution volumes used to calculate molecular weights were determined from triplicate injections.

## Supplementary Material

Appendix 01 (PDF)

Dataset S01 (XLSX)

Dataset S02 (XLSX)

## Data Availability

The sequences reported herein have been deposited to the GenBank database under the following accession numbers: *Cr*FoGS1 (PZ105964) (48), *Cr*FoGS2 (PZ105965) (49), *Cr*FoGS3-like (PZ105966) (50), *Ti*FoGS1 (PZ105967) (51), *Ti*FoGS2 (PZ105968) (52), *Ti*FoGS3 (PZ105969) (53), *Cr*HL1 (PZ105975) (54), *Cr*HL3 (PZ105976) (55), *Cr*MLP1 (PZ105970) (56), *Cr*MLP2 (PZ105971) (57), *Cr*MLP3 (PZ105972) (58), *Cr*MLP4 (PZ105973) (59), *Cr*MLP5 (PZ105974) (60). Datasets from VIGS RNAseq and Co-immunoprecipitations are included in supporting information. All other data are included in the manuscript and/or supporting information.

## References

[1] S. E. O’Connor, J. J. Maresh, Chemistry and biology of monoterpene indole alkaloid biosynthesis. Nat. Prod. Rep. **23**, 532 (2006). 16874388 10.1039/b512615k

[2] R. H. Himes, R. N. Kersey, I. Heller-Bettinger, F. E. Samson, Action of the vinca alkaloids vincristine, vinblastine, and desacetyl vinblastine amide on microtubules in vitro. Cancer Res. **36**, 3798–3802 (1976). 954003

[3] R. H. Himes, Interactions of the *Catharanthus* (*Vinca*) alkaloids with tubulin and microtubules. Pharmacol. Ther. **51**, 257–267 (1991). 1784631 10.1016/0163-7258(91)90081-v

[4] T. K. Brown, K. Alper, Treatment of opioid use disorder with ibogaine: Detoxification and drug use outcomes. Am. J. Drug Alcohol Abuse **44**, 24–36 (2018). 28541119 10.1080/00952990.2017.1320802

[5] K. R. Alper, H. S. Lotsof, C. D. Kaplan, The ibogaine medical subculture. J. Ethnopharmacol. **115**, 9–24 (2008). 18029124 10.1016/j.jep.2007.08.034

[6] H. Allain, D. Bentué-Ferrer, Clinical efficacy of almitrine-raubasine. Eur. Neurol. **39**, 39–44 (1998). 9516074 10.1159/000052069

[7] J. Achan , Quinine, an old anti-malarial drug in a modern world: Role in the treatment of malaria. Malar. J. **10**, 144 (2011). 21609473 10.1186/1475-2875-10-144 PMC3121651

[8] J. F. Treimer, M. H. Zenk, Purification and properties of strictosidine synthase, the key enzyme in indole alkaloid formation. Eur. J. Biochem. **101**, 225–233 (1979). 510306 10.1111/j.1432-1033.1979.tb04235.x

[9] A. Geerlings, M.M.-L. Ibañez, J. Memelink, R. Van Der Heijden, R. Verpoorte, Molecular cloning and analysis of strictosidine β-d-glucosidase, an enzyme in terpenoid indole alkaloid biosynthesis in *Catharanthus roseus*. J. Biol. Chem. **275**, 3051–3056 (2000). 10652285 10.1074/jbc.275.5.3051

[10] I. Gerasimenko, Y. Sheludko, X. Ma, J. Stöckigt, Heterologous expression of a *Rauvolfia* cDNA encoding strictosidine glucosidase, a biosynthetic key to over 2000 monoterpenoid indole alkaloids. Eur. J. Biochem. **269**, 2204–2213 (2002). 11985599 10.1046/j.1432-1033.2002.02878.x

[11] F. Trenti , Early and late steps of quinine biosynthesis. Org. Lett. **23**, 1793–1797 (2021). 33625237 10.1021/acs.orglett.1c00206 PMC7944568

[12] A. Stavrinides , Unlocking the diversity of alkaloids in *Catharanthus roseus*: Nuclear localization suggests metabolic channeling in secondary metabolism. Chem. Biol. **22**, 336–341 (2015). 25772467 10.1016/j.chembiol.2015.02.006 PMC4372254

[13] A. Stavrinides , Structural investigation of heteroyohimbine alkaloid synthesis reveals active site elements that control stereoselectivity. Nat. Commun. **7**, 12116 (2016). 27418042 10.1038/ncomms12116 PMC4947188

[14] E. A. Stander , The *Rauvolfia tetraphylla* genome suggests multiple distinct biosynthetic routes for yohimbane monoterpene indole alkaloids. Commun. Biol. **6**, 1197 (2023). 38001233 10.1038/s42003-023-05574-8 PMC10673892

[15] E. C. Tatsis , A three enzyme system to generate the *Strychnos* alkaloid scaffold from a central biosynthetic intermediate. Nat. Commun. **8**, 316 (2017). 28827772 10.1038/s41467-017-00154-x PMC5566405

[16] Y. Qu , Solution of the multistep pathway for assembly of corynanthean, strychnos, iboga, and aspidosperma monoterpenoid indole alkaloids from 19 E-geissoschizine. Proc. Natl. Acad. Sci. U.S.A. **115**, 3180–3185 (2018). 29511102 10.1073/pnas.1719979115 PMC5866588

[17] M. O. Kamileen , Conserved early steps of stemmadenine biosynthesis. J. Biol. Chem. **302**, 111120 (2025), 10.1016/j.jbc.2025.111120. 41478566 PMC12860956

[18] Y. Morita , A chalcone isomerase-like protein enhances flavonoid production and flower pigmentation. Plant J. **78**, 294–304 (2014). 24517863 10.1111/tpj.12469

[19] T. Waki , A conserved strategy of chalcone isomerase-like protein to rectify promiscuous chalcone synthase specificity. Nat. Commun. **11**, 870 (2020). 32054839 10.1038/s41467-020-14558-9 PMC7018950

[20] Z. Ban , Noncatalytic chalcone isomerase-fold proteins in *Humulus lupulus* are auxiliary components in prenylated flavonoid biosynthesis. Proc. Natl. Acad. Sci. U.S.A. **115**, E5223–E5232 (2018). 29760092 10.1073/pnas.1802223115 PMC5984530

[21] C. Li , Single-cell multi-omics in the medicinal plant *Catharanthus roseus*. Nat. Chem. Biol. **19**, 1031–1041 (2023). 37188960 10.1038/s41589-023-01327-0 PMC10374443

[22] S. Sun , Single-cell RNA sequencing provides a high-resolution roadmap for understanding the multicellular compartmentation of specialized metabolism. Nat. Plants **9**, 179–190 (2022). 36522449 10.1038/s41477-022-01291-y

[23] Y. Qu , Geissoschizine synthase controls flux in the formation of monoterpenoid indole alkaloids in a *Catharanthus roseus* mutant. Planta **247**, 625–634 (2018). 29147812 10.1007/s00425-017-2812-7

[24] I. Carqueijeiro , Alternative splicing creates a pseudo-strictosidine β-d-glucosidase modulating alkaloid synthesis in *Catharanthus roseus*. Plant Physiol. **185**, 836–856 (2021). 33793899 10.1093/plphys/kiaa075 PMC8133614

[25] S. C. Farrow , Biosynthesis of an anti-addiction agent from the Iboga plant. J. Am. Chem. Soc. **141**, 12979–12983 (2019). 31364847 10.1021/jacs.9b05999 PMC6706869

[26] J. Hwang , Ancient gene clusters govern the initiation of monoterpenoid indole alkaloid biosynthesis and C3 stereochemistry inversion. Nat. Commun. **16**, 10495 (2025). 41290611 10.1038/s41467-025-65543-z PMC12647599

[27] C. Langley , Expansion of the catalytic repertoire of alcohol dehydrogenases in plant metabolism. Angew. Chem. **134**, e202210934 (2022). 10.1002/anie.202210934 PMC9828224 36198083

[28] S. C. Carr, S. E. O’Connor, A tight-knit family: The medium-chain dehydrogenase/reductases of monoterpene indole alkaloid biosynthesis. Biochemistry **64**, 2712–2726 (2025). 40536199 10.1021/acs.biochem.5c00234 PMC12224309

[29] G. Guirimand , Strictosidine activation in Apocynaceae: Towards a “nuclear time bomb”? BMC Plant Biol. **10**, 182 (2010). 20723215 10.1186/1471-2229-10-182 PMC3095312

[30] A. S. Sandholu, S. P. Mujawar, K. Ramakrishnan, H. V. Thulasiram, K. Kulkarni, Structural studies on 10-hydroxygeraniol dehydrogenase: A novel linear substrate-specific dehydrogenase from *Catharanthus roseus*. Proteins **88**, 1197–1206 (2020). 32181958 10.1002/prot.25891

[31] L. Caputi , Structural basis of cycloaddition in biosynthesis of iboga and aspidosperma alkaloids. Nat. Chem. Biol. **16**, 383–386 (2020). 32066966 10.1038/s41589-019-0460-x PMC7104359

[32] L. Caputi , Missing enzymes in the biosynthesis of the anticancer drug vinblastine in Madagascar periwinkle. Science **360**, 1235–1239 (2018). 29724909 10.1126/science.aat4100

[33] B. Hong , Biosynthesis of strychnine. Nature **607**, 617–622 (2022). 35794473 10.1038/s41586-022-04950-4 PMC9300463

[34] B. K. Lombe , Biosynthesis of cinchona alkaloids. Nature **653**, 306–314 (2026). 41851462 10.1038/s41586-026-10227-x PMC13149305

[35] C. J. McClune , Discovery of FoTO1 and Taxol genes enables biosynthesis of baccatin III. Nature **643**, 582–592 (2025). 40500440 10.1038/s41586-025-09090-z PMC12240809

[36] S. Wang , Molecular mechanism underlying regulation of chalcone synthase by chalcone isomerase-like protein. Nat. Commun. **17**, 3992 (2026). 41832168 10.1038/s41467-026-70563-4 PMC13136490

[37] C. Wick , FoTO1 orchestrates Taxol biosynthesis through catalytic and non-catalytic mechanisms. bioRxiv [Preprint] (2026). 10.64898/2026.03.21.713420 (Accessed 24 March 2026).

[38] M. R. Park, X. Chen, D. E. Lang, K. K. S. Ng, P. J. Facchini, Heterodimeric O-methyltransferases involved in the biosynthesis of noscapine in opium poppy. Plant J. **95**, 252–267 (2018). 29723437 10.1111/tpj.13947

[39] N. Ozber , Alkaloid binding to opium poppy major latex proteins triggers structural modification and functional aggregation. Nat. Commun. **13**, 6768 (2022). 36351903 10.1038/s41467-022-34313-6 PMC9646721

[40] A. Casañal , The strawberry pathogenesis-related 10 (PR-10) Fra a proteins control flavonoid biosynthesis by binding to metabolic intermediates. J. Biol. Chem. **288**, 35322–35332 (2013). 24133217 10.1074/jbc.M113.501528 PMC3853281

[41] Y. Zhou , Protection of naringenin chalcone by a pathogenesis-related 10 protein promotes flavonoid biosynthesis in *Marchantia polymorpha*. New Phytol. **247**, 233–248 (2025). 40325841 10.1111/nph.70194 PMC12138178

[42] M. Gou, X. Ran, D. W. Martin, C.-J. Liu, The scaffold proteins of lignin biosynthetic cytochrome P450 enzymes. Nat. Plants **4**, 299–310 (2018). 29725099 10.1038/s41477-018-0142-9

[43] M. Boccia , A scaffold protein manages the biosynthesis of steroidal defense metabolites in plants. Science **386**, 1366–1372 (2024). 39418343 10.1126/science.ado3409

[44] M. Florean , A pseudoenzyme enables indole biosynthesis in eudicot plants. Nat. Chem. Biol. **22**, 120–127 (2025). 40562851 10.1038/s41589-025-01943-y PMC12727527

[45] S. C. Carr , Protein–protein interactions modulate a key branch point in monoterpene indole alkaloid biosynthesis. ACS Chem. Biol. **21**, 8–13 (2026). 41485246 10.1021/acschembio.5c00485 PMC12813968

[46] M. A. Digman, V. R. Caiolfa, M. Zamai, E. Gratton, The phasor approach to fluorescence lifetime imaging analysis. Biophys. J. **94**, L14–L16 (2008). 17981902 10.1529/biophysj.107.120154 PMC2157251

[47] J. R. Lakowicz, B. R. Masters, Principles of fluorescence spectroscopy. Third Edition. J. Biomed. Opt. **13**, 029901 (2008).

[48] S. C. Carr, S. E. O‘Connor, An auxiliary protein tunes reductase activity in alkaloid biosynthesis. GenBank. https://www.ncbi.nlm.nih.gov/nuccore/PZ105964. Deposited 3 March 2026.

[49] S. C. Carr, S. E. O‘Connor, An auxiliary protein tunes reductase activity in alkaloid biosynthesis. GenBank. https://www.ncbi.nlm.nih.gov/nuccore/PZ105965. Deposited 3 March 2026.

[50] S. C. Carr, S. E. O’Connor, An auxiliary protein tunes reductase activity in alkaloid biosynthesis. GenBank. https://www.ncbi.nlm.nih.gov/nuccore/PZ105966. Deposited 3 March 2026.

[51] S. C. Carr, S. E. O‘Connor, An auxiliary protein tunes reductase activity in alkaloid biosynthesis. GenBank. https://www.ncbi.nlm.nih.gov/nuccore/PZ105967. Deposited 3 March 2026.

[52] S. C. Carr, S. E. O‘Connor, An auxiliary protein tunes reductase activity in alkaloid biosynthesis. GenBank. https://www.ncbi.nlm.nih.gov/nuccore/PZ105968. Deposited 3 March 2026.

[53] S. C. Carr, S. E. O‘Connor, An auxiliary protein tunes reductase activity in alkaloid biosynthesis. GenBank. https://www.ncbi.nlm.nih.gov/nuccore/PZ105969. Deposited 3 March 2026.

[54] S. C. Carr, S. E. O‘Connor, An auxiliary protein tunes reductase activity in alkaloid biosynthesis. GenBank. https://www.ncbi.nlm.nih.gov/nuccore/PZ105975. Deposited 3 March 2026.

[55] S. C. Carr, S. E. O‘Connor, An auxiliary protein tunes reductase activity in alkaloid biosynthesis. GenBank. https://www.ncbi.nlm.nih.gov/nuccore/PZ105976. Deposited 3 March 2026.

[56] S. C. Carr, S. E. O‘Connor, An auxiliary protein tunes reductase activity in alkaloid biosynthesis. GenBank. https://www.ncbi.nlm.nih.gov/nuccore/PZ105970. Deposited 3 March 2026.

[57] S. C. Carr, S. E. O‘Connor, An auxiliary protein tunes reductase activity in alkaloid biosynthesis. GenBank. https://www.ncbi.nlm.nih.gov/nuccore/PZ105971. Deposited 3 March 2026.

[58] S. C. Carr, S. E. O‘Connor, An auxiliary protein tunes reductase activity in alkaloid biosynthesis. GenBank. https://www.ncbi.nlm.nih.gov/nuccore/PZ105972. Deposited 3 March 2026.

[59] S. C. Carr, S. E. O‘Connor, An auxiliary protein tunes reductase activity in alkaloid biosynthesis. GenBank. https://www.ncbi.nlm.nih.gov/nuccore/PZ105973. Deposited 3 March 2026.

[60] S. C. Carr, S. E. O‘Connor, An auxiliary protein tunes reductase activity in alkaloid biosynthesis. GenBank. https://www.ncbi.nlm.nih.gov/nuccore/PZ105974. Deposited 3 March 2026.

